# From high symmetry to high resolution in biological electron microscopy: a commentary on Crowther (1971) ‘Procedures for three-dimensional reconstruction of spherical viruses by Fourier synthesis from electron micrographs’

**DOI:** 10.1098/rstb.2014.0345

**Published:** 2015-04-19

**Authors:** Peter B. Rosenthal

**Affiliations:** Division of Physical Biochemistry, MRC National Institute for Medical Research, The Ridgeway, Mill Hill, London NW7 1AA, UK

**Keywords:** electron microscopy, cryomicroscopy, virus, reconstruction, image analysis, icosahedron

## Abstract

Elucidation of the structure of biological macromolecules and larger assemblies has been essential to understanding the roles they play in living processes. Methods for three-dimensional structure determination of biological assemblies from images recorded in the electron microscope were therefore a key development. In his paper published in *Philosophical Transactions B* in 1971, Crowther described new computational procedures applied to the first three-dimensional reconstruction of an icosahedral virus from images of virus particles preserved in negative stain. The method for determining the relative orientation of randomly oriented particles and combining their images for reconstruction exploited the high symmetry of the virus particle. Computational methods for image analysis have since been extended to include biological assemblies without symmetry. Further experimental advances, combined with image analysis, have led to the method of cryomicroscopy, which is now used by structural biologists to study the structure and dynamics of biological machines and assemblies in atomic detail. This commentary was written to celebrate the 350th anniversary of the journal *Philosophical Transactions of the Royal Society*.

## 1. Introduction

Early members of the Royal Society, notably Robert Hooke and Antonie van Leeuwenhoek, invented new designs for the light microscope and applied them to imaging a previously unseen world of tiny creatures. Since then, the microscope has been one of the most powerful tools for extending the reach of the senses in both the life sciences and physical sciences. On a much smaller scale, the visualization of viruses, the causative agents of disease in humans, animals and plants, awaited the twentieth century development of the electron microscope, which can illuminate specimens with radiation of wavelength more than 100 000 times smaller than that of visible light. Viruses are assemblies that package their genetic code as nucleic acid, either DNA or RNA, for transfer from a host cell to their next target of infection. The electron microscope showed that while some types of viruses were variable in structure, others were remarkably uniform, either as rod-shaped or spherical particles. Structural studies of viruses and viral proteins by electron microscopy and X-ray crystallography have complemented biochemical and genetic approaches in understanding the life cycle of viruses, a subject important to both basic science and medicine.

R. A. Crowther's ‘Procedures for three-dimensional reconstruction of spherical viruses by Fourier synthesis from electron micrographs’ [1] described the first objective computational approach to determine the three-dimensional structure of a spherical virus particle from images. By combining electron images of identical, symmetric virus particles in different orientations, the paper describes the first ‘single particle reconstruction’, a term now applied to a method for structure determination of macromolecular assemblies. The paper was presented at a symposium on ‘New developments in electron microscopy’ at the Royal Society on 13 March 1970 organized by Hugh Huxley and Aaron Klug. These new developments were reported in three areas: (i) instrumentation, (ii) sample preparation, and (iii) image analysis. Early microscopy pioneers of the Royal Society would have emphasized progress in only the first two areas. Crowther's paper was a contribution in the third area of development and was an application of the new electronic computers of the era to extracting biological information from electron micrographs. In the subsequent 45 years, continued developments in these three areas have led to a general and widely applied method for structure determination of large macromolecular assemblies. Dramatic progress in the last couple of years has extended its application to atomic resolution studies of smaller asymmetric proteins and nucleic acid assemblies, providing biology with one of its most powerful tools for imaging molecular structure. It is therefore an instructive moment to consider the origins of single particle reconstruction as well as both the incremental steps and leaps that have brought electron microscopists and structural biologists to the present moment of excitement and optimism.

## 2. Electron microscopy and virus structure

The studies described by Tony Crowther in *Philosophical Transactions* and in related publications with colleagues were performed at the MRC Laboratory of Molecular Biology (LMB) in Cambridge, where during the course of his career he was a graduate student, research group leader and Joint Head of the Structural Studies Division. The LMB has an illustrious history of achievement in macromolecular studies including X-ray crystallography, the first method to reveal the atomic structure of proteins, and many of the developments in electron microscopy discussed in this essay happened there. In 1972, around the time of the spherical virus work, Crowther described the fast rotation function in X-ray crystallography, a procedure for finding the orientation of protein models in crystals [2]. Progress in X-ray crystallography and electron microscopy occurring at the same time and under the same roof reflects among other things the similar goals and underlying physical and mathematical principles of the two techniques.

**Figure 1.**
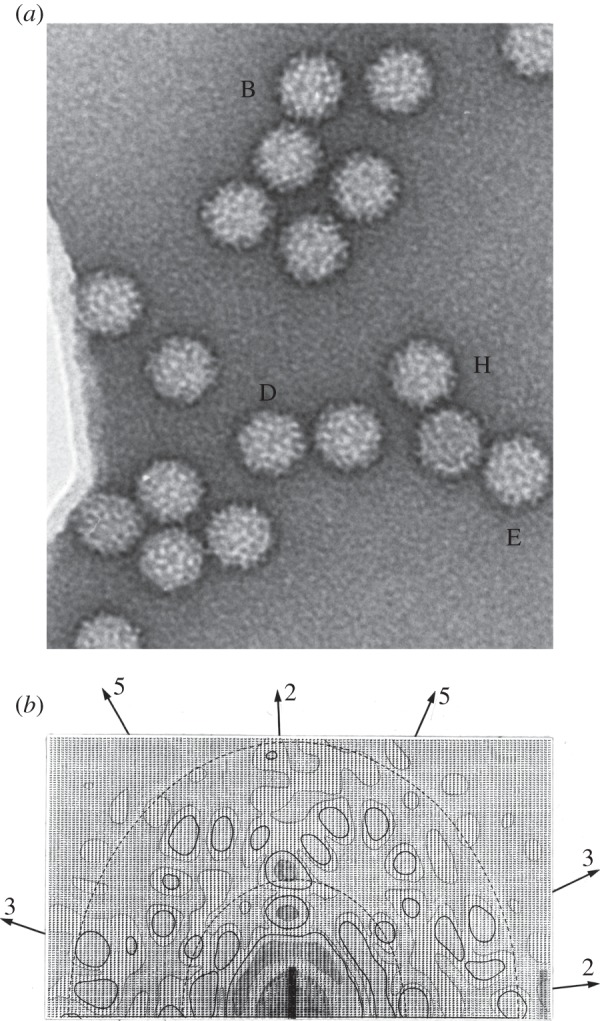
(*a*) Glass plate image from Crowther [1] of negatively stained TBSV particles taken by John Finch. (*b*) Map of the amplitudes of a two-dimensional Fourier transform of a TBSV particle (particle B in *a*) viewed down the icosahedral twofold axis, with directions of additional twofold, threefold and fivefold symmetry axes indicated. Original line printer output. Source for fig. 2 of original manuscript [1], kindly provided by R. A. Crowther.

Tomato bushy stunt virus (TBSV) is a small, spherical virus that has featured as a prototypical macromolecular assembly in several landmark studies in structural biology, including Crowther's first work on virus reconstruction [1,3,4]. In the electron microscope, the virus particles appear as highly uniform spheres with surface features suggesting polyhedral symmetry (figure 1*a*). Purified TBSV particles are so regular that they form well-ordered three-dimensional crystals with cubic-shaped unit cells implying that each virus particle is identical in structure. In 1956, Crick & Watson [5] summarized both structural and chemical evidence that spherical shells or ‘capsids’ of viruses such as TBSV contain multiple copies of a single kind of subunit with a regular packing, and because of this definite geometry, a plant virus could be considered a molecule. They argued that using many copies of a single protein to build the largest regular structure possible solves the problem of packaging the nucleic acid genome with a minimum of genetic information. The largest such regular structure is the icosahedron, a Platonic solid with 20 triangular faces and 60-fold symmetry. In the same issue of *Nature*, Don Caspar's X-ray photograph of a slice of the three-dimensional diffraction pattern of a TBSV crystal showed intensity spikes indicating an arrangement of fivefold, threefold and twofold symmetry axes consistent with the symmetry of the icosahedron [6].

During the 1960s Klug, Finch and co-workers studied the architecture of virus capsids by imaging viruses in the transmission electron microscope using negatively stained specimens [7]. In the negative stain method, biological specimens, normally found in an aqueous environment, are embedded in a thin layer of dried heavy metal salt, a preparation that is stable in the vacuum of the electron microscope. Because biological specimens are composed of light atoms, the structures appear in high contrast compared with the background stain, which scatters electrons strongly. This staining revealed the envelope of the particles at relatively low resolution, and though there could be distortions of the particle and other artefacts, the preparation method was adequate to see morphological features. These studies elaborated Caspar & Klug's theory of quasi-equivalence [8] which describes how capsids containing multiples of 60 subunits (e.g. 180 for TBSV) can be organized with icosahedral symmetry.

Their structural studies of viruses in stain also focused on a more detailed understanding of how the electron images were formed. By comparing images of virus particles that were sitting in stain on only one side versus those that were stained on both sides, they learned that images of virus particles uniformly embedded in stain were super-positions of the top and bottom of the capsid shells [9]. This is a consequence of the very large depth of field of electron lenses, which means that an electron image is a projection of the structure. By comparison, confocal light microscopes have nearly as good vertical resolution as in-plane resolution, so three-dimensional information about the specimen may be obtained by focusing at a series of planes through the specimen. In an electron micrograph, overlap of information from different levels in the specimen causes difficulty in interpreting the image. The images can therefore only be understood in terms of the three-dimensional structure that gives rise to the projections. The first approach to interpreting the images was to build three-dimensional physical models and make shadow projections of the models with light [10,11] and even more elaborate analogues for negative stain images [12].

Further insight into projection images in the electron microscope came from the analysis of rod-shaped viruses, such as tobacco mosaic virus (TMV), understood to be a helical organization of identical protein subunits that assemble around its RNA genome [5]. Owing to this periodic structure, an electron micrograph of an individual helical virus particle acts like a diffraction grating and laser diffraction was used as a method to analyse structure [13,14]. Subsequently, a new technology, a computer-controlled film densitometer (programmed by Crowther, then a graduate student) [15] was used to read the optical density at steps along an electron micrograph and store the data as a rectangular array of numbers (pixel values). The calculation of diffraction patterns could be performed on the digitized image in the computer by Fourier transformation, a mathematical technique in which an image or a volume is analysed in terms of its spatial frequencies. The Fourier transform of an image of a TBSV particle (figure 1*b*) shows strong intensities along the directions of the symmetry axes, consistent with Caspar's [6] observations.

Protein crystallographers had learned that the diffraction patterns recorded while illuminating different orientations of a crystal were portions of a single three-dimensional lattice or diffraction pattern. The three-dimensional diffraction pattern is related to the structure of the protein in the crystal through the mathematics of the Fourier transform. There is a direct mathematical or computational procedure to determine the structure of a molecule from its three-dimensional Fourier transform called Fourier inversion.

In the years immediately before Crowther's work on spherical viruses, a breakthrough occurred in the interpretation of projection images from the electron microscope through Fourier analysis, enabling the direct calculation of the three-dimensional structure or ‘reconstruction’ of the helical tail of T4 bacteriophage [16]. The interpretation of a projection image depends on the Fourier projection/section theorem: the Fourier transform of a two-dimensional projection of an object is equivalent to a central slice through the three-dimensional Fourier transform of the object. The Fourier transform of the T4 tail image is a single slice through the three-dimensional transform, but an important consequence of the helical symmetry is that all the slices about the helical axis are then known. Thus, for the special case of a helical specimen, an image of one particle is all that is required to calculate its three-dimensional structure, at least to low resolution. In concluding their work, DeRosier & Klug proposed a general principle for the reconstruction of non-helical particles in which data for all the required projected views would be recorded and combined [16].

## 3. Three-dimensional reconstruction of a spherical virus

At the time of the Royal Society meeting, Crowther *et al*. [4] had submitted an article to *Nature* describing reconstructions of both TBSV and human wart virus. The paper presented at the Royal Society meeting was a description of new methods for reconstruction with TBSV as an application. Crowther acknowledged Linda Amos, with whom he co-authored several papers at this time, for writing computer programs for the new procedures. Two approaches were used for recording the views required for reconstruction. In the case of the human wart virus, two views of each particle were obtained by tilting the specimen holder through a known angle, a forerunner of the multiple tilts used to collect data for tomography of non-symmetric specimens [17]. In the case of the TBSV particles, images of several different particles were used, with the stated advantage that individual particle imperfections would be averaged out.

The non-helical, spherical virus specimen presented a new problem. Images corresponding to different projections of the virus need to be combined in one reconstruction. This requires determining the orientation of different virus particles within the images and centring them in a single coordinate system. The specimen was a good candidate because the virus has distinguishable features in negative stain, and earlier evidence strongly indicated that it has icosahedral symmetry. A simple formula described the minimum number of views required to calculate a structure of a given diameter to a desired resolution. If an image could be assigned an orientation, then by application of the symmetry of the virus many more views of the virus could be calculated. Because the Fourier transform of an image represents a slice through the three-dimensional Fourier transform of the virus, then the three-dimensional Fourier transform can be filled up by a few images (five for the TBSV study), each with up to 59 symmetry-related views. The structure of the virus could then be calculated by an inverse Fourier transform as with crystals or helical specimens.

Thus, the key issue in the analysis of single particles is to assign the orientations. Crowther observed that any two slices of the three-dimensional Fourier transform have a geometric intersection through a line where each transform would have the same values, referred to as a ‘common line’. In principle, the computer can search all the lines in two slices to find the common line, but this is very difficult for noisy, imperfect images. However, the symmetry of the virus can be exploited: one common line implies the existence of other common lines within a single image. A search for the number and location of many ‘self-common lines’ uniquely identifies the orientation of the icosahedral symmetry axes of the particle observed in the projection. The best assignment of a particle's orientation is one that gives the greatest numerical agreement between all common lines predicted for that orientation. Approximate values for the orientation and position of the particle in the image can be further improved by ‘refinement’. Several refined images can thus be consistently oriented in the same coordinate system.

**Figure 2.**
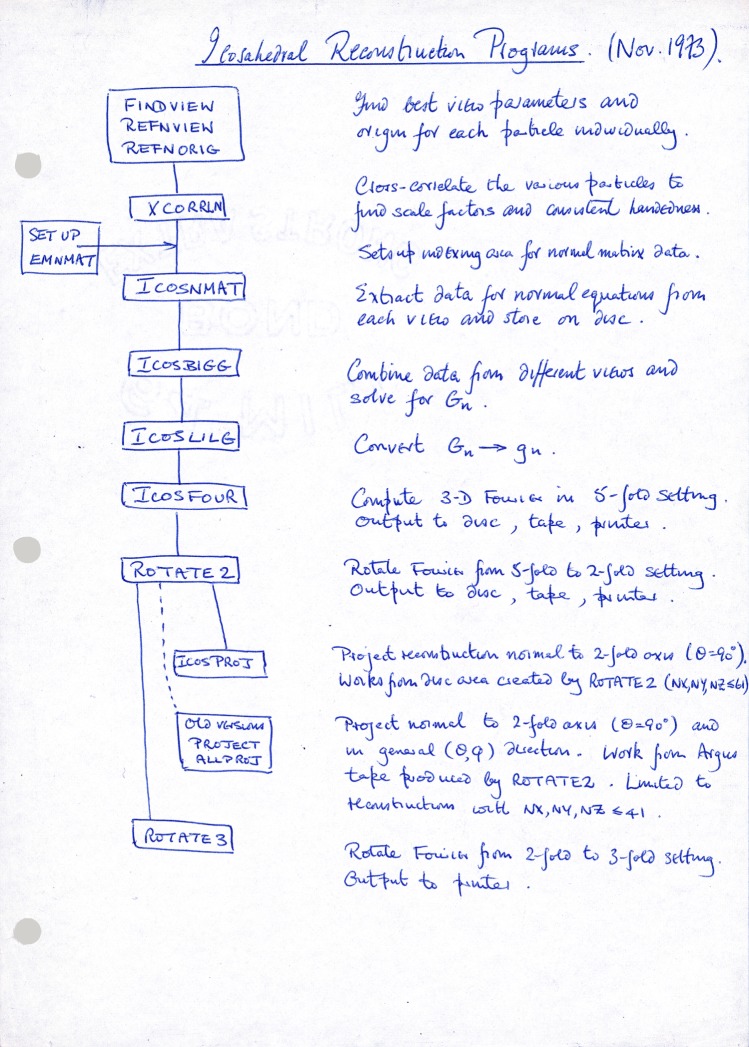
Flowchart of the icosahedral reconstruction procedure written by Crowther in 1973. Kindly provided by R. A. Crowther.

A number of significant computational issues needed to be solved to calculate the structure from separate projection images, including making sure that the data are sampled finely and regularly onto the final grid or volume on which the structure is calculated and presented. Some of the issues were discussed in an earlier work of Crowther *et al*. [18]. The method of calculation using a cylindrical polar coordinate system was an efficient solution with consideration of the available programs and computing power at the time. Modern programs that process all types of single particle specimens use a Cartesian coordinate system. Figure 2 shows Crowther's handwritten flowchart for the procedure.

**Figure 3.**
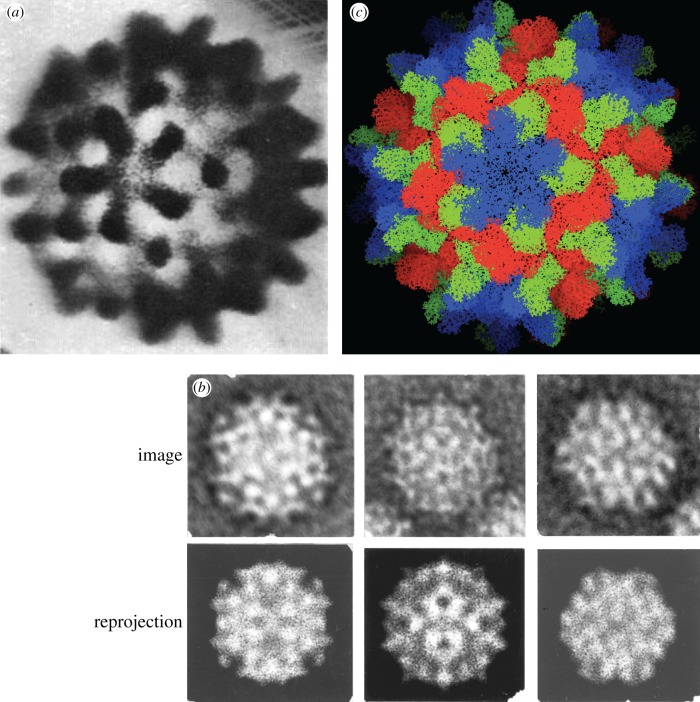
(*a*) Original reconstruction of TBSV from five particles reported in the 1971 paper. (*b*) Particle images and corresponding reprojections of the TBSV reconstruction show agreement. (*c*) High-resolution atomic model of TBSV determined by X-ray crystallography in 1978. Images provided by R. A. Crowther.

The map of the virus calculated in this way (figure 3*a*) showed how 90 morphological units, each explained as a dimer of the 180 capsid monomers in the virus, were arranged with respect to the icosahedral symmetry elements as well as local symmetry elements. The paper contained, for the first time, several recognizably modern demonstrations of the success or reliability of the reconstructions. Calculated projections of the reconstruction agreed with the experimental images (figure 3*b*). The paper gives a measure of the reconstruction quality (the agreement between matched common lines) as a function of resolution and showed a correlation to the expected resolution limit of negative stain (approx. 30 Å). This is sufficient to confirm the assumption of icosahedral symmetry and that the alignment methods are working. The modern expert will also recognize a number of other familiar features such as the enforcement of a consistent absolute hand and magnification as determined from the ‘cross common lines’ between particles.

In short, the paper had accomplished three-dimensional image reconstruction from two-dimensional images of non-crystalline single particles. Spherical viruses are an ideal model system for developing methods, and though not the most general specimen, they are an important generalization from helical or crystalline specimens. Because of the limitations of the large grain size of negative stain and the small number of images, the structure of TBSV could only be determined to very low resolution. The paper was an important beginning to the single particle reconstruction field and a glimpse of the road ahead. While Fourier-based methods have been widely developed and applied, alternative iterative approaches to structure determination from projections in real space [19,20] have proved useful in some contexts. Fourier methods are also the natural way to treat microscope aberrations, an essential part of high-resolution image analysis and the subject of another paper presented at the meeting in 1970 [21]. In addition to Crowther's original programs, modern variations of the procedure are used by those active in structure determination of icosahedral viruses [22–24]. The continued relevance of the methods, at least in outline, and the clarity of their presentation make Crowther's paper worthwhile reading for a student today.

The atomic model for TBSV, the first high-resolution view of a crystalline virus, was determined at 2.9 Å by Stephen Harrison and co-workers using X-ray crystallography [25] (figure 3*c*). The structure showed how the capsid protein uses two flexibly linked domains to adopt the different conformations required by quasi-equivalent packing. Radiation-sensitive virus crystals, many weak reflections and the difficulty of solving the crystallographic phase problem for a large molecular assembly posed huge technical challenges and led to important new methods in crystallography. Yet, the symmetry of the virus posed constraints on the density that were used to solve the structure. The properties of TBSV, its regularity and symmetry, were exploited in both electron and X-ray studies. Thus, TBSV and other spherical viruses helped to drive the development of ideas and methods in both fields [26–28]. In the two subsequent decades, X-ray crystallography remained the widely applied high-resolution technique while electron microscopy was applied to lower-resolution studies of large assemblies. Very few would have thought that eventually electron microscopy could routinely reach atomic resolution.

## 4. The development of high-resolution single particle electron cryomicroscopy

Progress in the field of three-dimensional electron microscopy resulted from many developments in each of the three areas described at the 1970 Royal Society meeting. As anticipated in the conclusion of Crowther's paper, structure determination from individual particle images could be extended to particles with lower symmetry or even to proteins with no symmetry at all. For an asymmetric particle, many more views are required and the specific methods employed by Crowther for determining orientations could not be applied. Furthermore, images of particles in negative stain consist of desirable signal from the particle as well as noise that comes from the underlying structure of the carbon film support, particle distortion, stain variation and the photographic process. But by averaging similar views of a particle, the common signal of the particle in the images is increased compared with that of the noise, resulting in a higher signal-to-noise image. The ribosome, a large asymmetric macromolecular assembly of central biological importance as the engine of protein synthesis, was an excellent target for electron microscopy and drove the development of methods for studying asymmetric particles. The key developments included methods for statistical classification and alignment of similar views of the particles so that many high signal-to-noise averages of the particle can be obtained [29]. In addition, a method for determining the relative orientations of the views of an asymmetric specimen for subsequent three-dimensional map calculation was required. The first successful strategy was a two exposure method called ‘random conical tilt’, which assigns orientations to particles in an image field recorded when the specimen is tilted, provided that the same particles all display a similar view to each other when the specimen is untilted (usually due to a preferential interaction of the particle with the carbon) [30]. A computational procedure called angular reconstitution for aligning images based on real space common line projections with similarities to Crowther's Fourier transform methods also succeeds in determining the relative orientation of high signal-to-noise particle views but without requiring tilting of the specimen [31]. A map achieved by either method can be used to calculate projections that can then be matched against experimental views, thus assigning the experimental view an orientation. An iterative process of model-based refinement improves the assignment of orientations and the quality and resolution of the resulting map. These methods were later applicable to the frozen-hydrated specimens studied by electron cryomicroscopy (cryoEM) [32]. Progress on negative stain specimens, however, was destined to reach a limit, providing only low-resolution molecular envelopes. New experimental approaches were required for studies at high resolution such as those obtained by X-ray crystallography.

High-resolution structure determination by electron microscopy was first demonstrated for bacteriorhodopsin (BR), the light-driven proton pump that forms crystalline patches in the purple membrane of *Halobacterium halobium*. In 1975, Henderson & Unwin [33] calculated a three-dimensional map at 7 Å resolution from two-dimensional BR crystals showing the trans-membrane α-helices of a membrane protein for the first time. A two-dimensional crystal is a periodic array of molecules one molecule thick in which each is held in fixed position and orientation as determined by unit cell dimensions and symmetry of the crystal. Different views of the molecules, each corresponding to a slice through the three-dimensional Fourier transform are obtained by tilting the specimen. Fourier transforms of their images reveal a crystalline lattice, and their study is sometimes called electron crystallography, but the images provide both amplitudes and phases, unlike X-ray diffraction patterns, which give only amplitudes.

The work reported several major methodological advances. They studied unstained specimens in glucose, which effectively replaced the water in the sample but preserved native protein structure to high resolution. The resulting maps of the protein show density variations in the structure of the protein itself, including its interior, rather than the stain distribution. Such thin biological specimens are composed of light atoms and require small amounts of defocus during imaging to enhance their contrast, typically between a few hundred nanometres and up to a few micrometres. Furthermore, biological specimens without stain are extremely sensitive to irradiation by the electron beam, more so than the negative stain preparations discussed so far. The fading of the crystalline patterns of BR showed that images should be recorded with less than five electrons impinging on a square Ångström of the specimen to preserve high-resolution features. These studies therefore employed imaging methods that minimized the exposure of each crystalline specimen to a single ‘low dose’ image. For low dose images, statistical variation in electron counting is a major source of the noise. The diffraction patterns calculated from the images are the computed average of the scattering of all the molecules in the crystal. Analysis of the patterns is similar to averaging images of all the individual molecules in the crystal, thus overcoming statistical noise and giving a high-resolution image of the molecule.

In subsequent years, Henderson and colleagues developed experimental and computational methods for analysing two-dimensional crystalline specimens to high resolution, ultimately obtaining a map for BR at 3.5 Å resolution, sufficient to build an atomic model [34]. Data collection on specimens held at liquid nitrogen temperature (−190°C) is critical for reducing radiation damage and recording high-resolution images. Important instrument developments during this time were computer control of the microscope for low dose image acquisition and field emission gun microscopes which provide a more coherent source of radiation than earlier thermionic electron guns [35]. Coherence is critical for recording high-resolution information and for calculating the effect of microscope aberrations and the focal setting in the image, parameters that are necessary for extracting high-resolution structural data from electron micrographs. Although near atomic resolution structures have been demonstrated for several other two-dimensional crystals, they remain a specialized specimen type, powerful for membrane proteins, but were important for developing experimental and computational methods and showing what was possible with electrons.

The breakthrough in sample preparation described by Jacques Dubochet and co-workers, following earlier attempts to prepare frozen-hydrated specimens [36], provides a general method for sample preparation that preserves structure to high resolution [37]. This could be applied to a wide range of specimen types for imaging by ‘cryoEM’ from single particle specimens to whole cells. A thin film of the specimen in buffer is plunged into a cryogen, such as liquid ethane, cooling it rapidly before ice crystals can form, and creating the near native environment of amorphous ice. The thin film remains stable in the microscope vacuum when held at liquid nitrogen temperature. A wide range of specimens including Semliki Forest virus [38,39] were frozen in random orientations in thin films over holes, avoiding the granular noise contributed by the carbon support. When low dose procedures are applied to such specimens, the resulting images are noisy, but have the aforementioned advantages of an unstained specimen. A single high-resolution image of a field of identical particles in random orientations can be recorded. However, averaging of different identical particles in the same orientation reinforces the signal over the noise, creating higher contrast views of the particle for reconstruction. By applying image processing methods such as described for the ribosome, a wide range of specimens were studied, but mostly at a resolution worse than 10 Å.

The near atomic resolution information obtained from two-dimensional crystals and some helical specimens showed that high-resolution protein structure determination was possible with electrons, and the number of molecules in the crystalline lattice gave an indication of how many images of individual, unstained protein molecules would be required to reach atomic resolution. However, the molecules in crystals have the benefit of being aligned to each other, and their orientation is known from the easily measurable orientation of the lattice. In single particle analysis, each molecule's position and orientation must be determined in the image. The ability to find the orientation of a particle depends on whether it is large enough to scatter electrons strongly compared with the background noise, i.e. how visible it is. A theoretical calculation showed that for images limited only by the noise associated with low electron dose, 10 000 particle images should be sufficient to reach atomic resolution (3 Å) for a single particle of any size [40], though the lower size limit was approximately 100 kDa. At the same time, quantitative examination of the amount of contrast in cryoimages showed that they were imperfect due to a number of factors including movement of the specimen, sometimes induced by the electron beam itself. Movement of the specimen effectively blurs the image and limits the higher-resolution information [41]. The consequence of image imperfections is that many more particles are required to reach high resolution, even for a relatively large complex. Perhaps even millions. And smaller objects cannot be aligned reliably at all. The calculation stimulated efforts to reach high resolution by showing that relatively few particles would be required if some of the experimental and theoretical problems could be solved.

**Figure 4.**
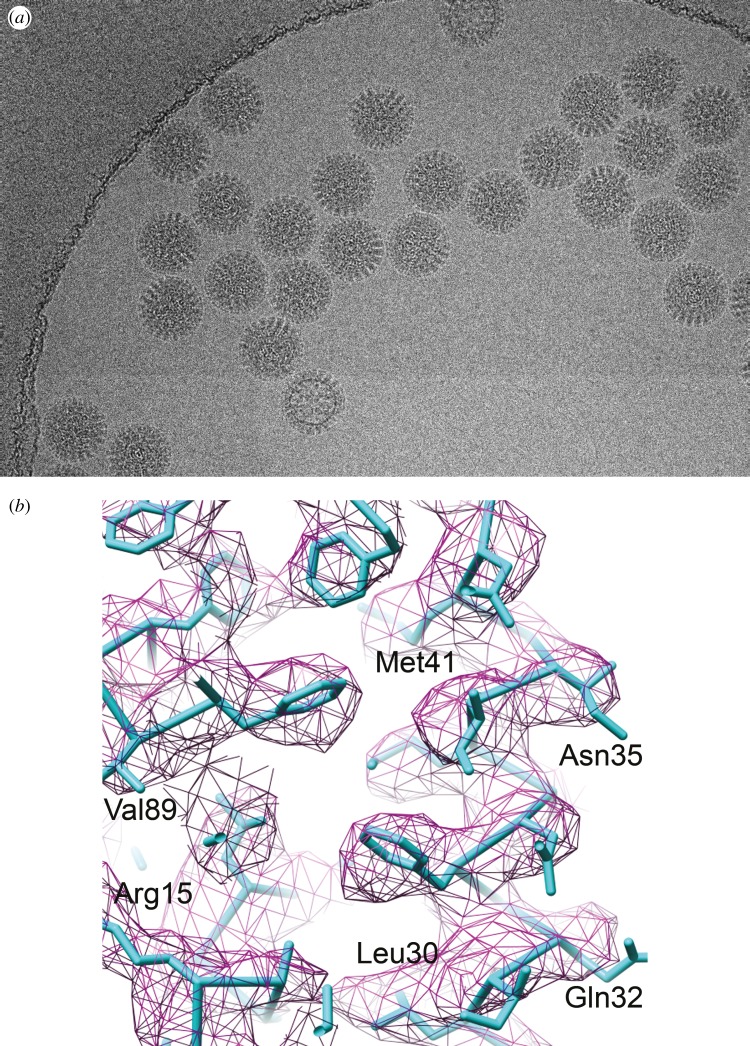
Structure of 50 MDa rotavirus capsid based on cryoEM at 3.5 Å resolution. (*a*) Low dose image of frozen-hydrated particles. (*b*) Close-up of map density for protein showing side chains and amino acid sequence labels. Adapted from [44]. Copyright © The National Academy of Sciences.

Instruments, sample preparation and image analysis were now poised for high-resolution studies of single particles. Symmetric viruses were the most likely to get there first because they are highly visible in amorphous ice due to their large size and are therefore easier to align. Aligning and averaging icosahedral particles includes 60 times more monomers or ‘asymmetric units’ than for an asymmetric particle. An important milestone was achieved by imaging a virus sub-particle, the icosahedral capsid assembly of hepatitis B virus, published in *Nature* by two groups [42,43]. In the study by Böttcher & Crowther, 6400 virus particles were averaged (384 000 asymmetric units when 60-fold symmetry is taken into account) to obtain a three-dimensional map at 7.5 Å resolution. The map revealed the fold of the polypeptide chain in the monomer and allowed the approximate assignment of amino acids positions, the first time this had been achieved for a non-crystalline specimen. This was a big leap from the five particles presented in Crowther's original description of the methods. Common lines were again the basis for image analysis of the particle, but this study included ‘cross common lines’ as a means of matching projections of a model with images [23]. The calculations required a huge amount of computer time for the refinement of particle orientations and correction for microscope aberrations necessary to bring out small details of the structure. About 10 years later, the high-resolution structures of the rotavirus double-layered capsid particle (50 MDa) [44] shown in figure 4 and the infectious sub-virion of aquarevious (72 MDa) [45] presented icosahedral particle maps that were interpretable by atomic models. This means the trace of the polypeptide chain through the map could position the amino side chain positions with certainty. These maps required about 10 000 icosahedral particles. Maps of smaller particles were improving but they did not approach atomic resolution.

For many years, single particle images have been recorded on photographic film, which captures high-resolution detail, and, for example, was the recording medium in the atomic resolution virus studies mentioned above. When electronic detectors (phosphor-based CCD cameras) became available, they were used widely because of their speed and convenience, even though film was a better detector. In the last couple of years, a new generation of direct electron detectors (DDD's or Direct Detector Devices) has been applied to imaging single particles, producing better images than film [46]. In addition to improving image quality at high resolution, the new detectors can record many images per second, thus capturing the image of a particle over many frames, recording a movie of the specimen. These revealed the movement of the specimen during the course of a low dose image. Suddenly, a new opportunity for image analysis arose. By computationally aligning the particles between frames, a new image that is corrected for the movements is obtained [47–49]. In addition, the frames that contain high-resolution data can be chosen for analysis, and the frames where the specimen shows damage discarded. The new structures calculated from these movies showed dramatically improved high-resolution features and a ‘resolution revolution’ was proclaimed [50].

**Figure 5.**
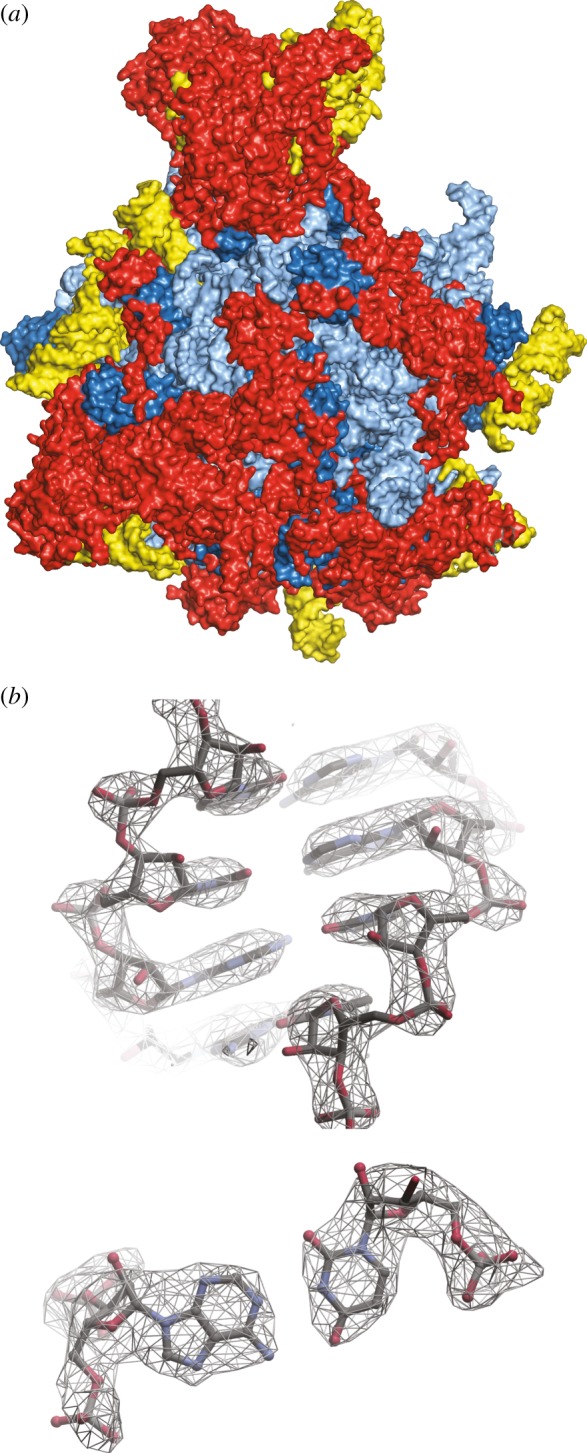
(*a*) Structural model based on cryoEM for the 54S yeast mitochondrial large ribosomal subunit, an asymmetric particle of mass 1.9 MDa, at 3.2 Å resolution. (*b*) High-resolution density for RNA bases. Adapted from [51]. Reprinted with permission from the American Association for the Advancement of Science.

The asymmetric 1.9 MDa 54S subunit of the ribosome, much smaller than the viruses described at high resolution, could now be solved close to 3 Å by electron microscopy and from fewer particles than previously imagined [51] as shown in figure 5. This resolution improvement is critical because, as learned from years of X-ray analysis, it is then possible to extend from a secondary structure model to an accurate placement of amino acid side chains or nucleotide bases, positioning the chemical groups that are the basis for the molecular mechanism. The tetrameric TRPV1 ion channel, a 300 kDa membrane protein complex, was determined to 3.5 Å in some regions [52]. The current state of the field is represented by the 4.5 Å structure of γ-secretase, a membrane protease that is responsible for the aberrant products that form amyloids associated with Alzheimer's disease [53]. Though achieving slightly lower resolution than the other studies, γ-secretase is an asymmetric molecule of about 170 kDa. While cryomicroscopists previously chose projects that were suited to the technique, there are now manifold specimens in this lower molecular weight range for which microscopy will be a fair competitor to crystallography.

## 5. High-resolution structure determination by electron cryomicroscopy: the future

It would appear at the time of writing that many of the major obstacles to the atomic resolution imaging of non-crystalline biological specimens by cryoEM have now been surmounted, some of them in the last year or so. Electron microscopy is a technique with general applicability to a range of specimen types, including single particles, fibres and two-dimensional crystals. One hundred years after Bragg's Nobel Prize for the development of X-ray diffraction analysis, single particle cryoEM joins X-ray crystallography and NMR spectroscopy as a high-resolution structural method for biological macromolecules. Because of its similar goal to X-ray crystallography, the single particle approach has been described as crystallization *in silico* because the alignment and averaging of images is done by the computer and no longer requires the growth of well-ordered crystals of the specimen. Microscopists have not yet reached atomic resolution for the lowest molecular weight limit or smaller number of particles predicted by theory [40], but avenues for improvement have been identified and may push the method toward much lower molecular weight particles. These improvements may include new detectors that count individual electrons and specimen supports that reduce movement.

Future developments of three-dimensional electron microscopy with impact in biology will include further generalization of the types of specimens that can be imaged at high resolution. Even with recent rapid advances, high-resolution single particle analysis is largely restricted to proteins that are biochemically purified and are assumed to be nearly identical in structure. In the future, electron microscopy will include many more investigations where these assumptions can be relaxed to include specimens with structural flexibility and heterogeneity. The function of many molecular machines is achieved through large structural transformations. The classical approach is to trap the molecules in a well-populated intermediate for imaging. But by breaking the molecular machines out of crystalline lattices, we can hope to image the structural transformations happening in solution. Examples include the membrane rotary ATPases, whose motion is linked to the synthesis or hydrolysis of ATP [54], and the chaperonin GroEL/ES [55], which assists the folding of proteins.

Of course, when a specimen has conformational or compositional heterogeneity, it is more complex to tell a computer how to classify and average particle images. Any particle image can be assigned to one of several different structures and to different orientations. Each member of the ensemble of structures requires an independent structure determination and many more particle images are required for the whole multiple structure dataset. Statistical approaches to image processing such as maximum likelihood have contributed to the resolution revolution [56], but at the same time are probably in their infancy. Better quality images will always help when dealing with problems of increasing structural complexity as will large datasets from automated data collection, but we shall have greater challenges in analysing them. As cryoEM becomes more useful, it will also become a technique applied by the non-specialist, in which case it may be the computer that arrives automatically at the answer to a complex problem. Therefore, computational methods that can be applied reliably will require continued development. Crowther and co-workers in several ways addressed the question of whether their TBSV structure was correct. Validation methods [57–59] that prevent computers from refining an incorrect model against noisy data [60] must continue to be developed as cryoEM is applied to the most challenging specimens.

While symmetrical viruses can be solved by single particle methods, many viruses that are important human pathogens are variable in structure or pleomorphic, such as the lipid-enveloped influenza viruses. Each virus is different, and images of different viruses cannot be averaged together, and they are best studied by cryotomography. In tomography, all the unique projection images required to determine the structure by electron microscopy are recorded from a single specimen as a tilt series, and the resulting three-dimensional reconstruction is called a tomogram [61]. There is an advantage over single particle reconstruction in that the relative angles of the images are known from the experiment and they do not require de novo alignment. However, the single specimen receives a larger electron dose, and accumulated radiation damage places a limit on the resolution of the resulting structure. Nevertheless, the low-resolution maps obtained by cryotomography of influenza virus [62] may be interpreted, for example, with high-resolution X-ray models for the surface glycoproteins, the haemagglutinin [63] and the neuraminidase [64], creating pseudo-atomic models of the virus. Higher-resolution molecular structures are possible through tomography by aligning and averaging tomogram sub-volumes containing identical or similar molecules. Sub-tomogram averaging for a helically ordered specimen has reached sub-nanometre resolution [65], an important threshold for visualizing the fold of a protein. On a larger scale, one of the most exciting endeavours is the high-resolution structural study of cells by cryotomography with the goal of imaging proteins and their assemblies *in vivo* [66].

Where molecular averaging is not possible, there may still be opportunities for higher-resolution studies through improved instruments and improved image analysis. The defocus mechanism of contrast used in cryoEM at present is imperfect because the contrast is not uniform across all resolution ranges. Zernike's phase plate for the light microscope had been proposed by 1940 and has been used in light microscopy for many years. Phase plates for electron microscopy are currently an active area of research and could help record high-contrast, in-focus images. Interestingly, the *Transactions* published a paper presented by Nigel Unwin at the 1970 Royal Society meeting that contained a description of a phase plate consisting of a thin spider web coated with metal suspended across the objective aperture of an electron microscope, and this produced phase contrast images of TMV [67]. Though better imaging methods and calculations may allow us to get more information for a given electron dose, radiation damage to the specimen is ultimately the limiting factor for imaging with electrons and X-rays. It is a question for the more distant future if there are approaches to atomic resolution imaging of radiation-sensitive biological specimens that can beat the radiation damage problem. One proposal is to record images or diffraction patterns extremely rapidly (within femtoseconds) with a pulsed radiation source so that the structure of the specimen is recorded before it can react to the damage [68].

There has been a history of improvement in the instrumentation used to image biological macromolecules while the means to record the image and analyse its features has similarly undergone dramatic changes. Robert Hooke's spectacular drawings in *Micrographia* tell us what he saw through the eyepiece. The development of camera technology and computational methods in microscopy has allowed a more detailed and objective analysis of the specimen. In electron microscopy, image analysis revealed molecular structure by first exploiting the special features of crystalline and helical specimens and then finding ways to analyse less periodic specimens, as exemplified by Crowther's paper. Advances with prototypical specimens revealed how instrumentation, sample preparation, data collection and analysis could be improved to solve the structure of the most general, asymmetric specimens. Our ability to collect vast image datasets and analyse them rapidly by computer will no doubt extend our understanding of biological structure and function. Images are now just frames in a movie of the specimen's lifetime. Computational image analysis has become an essential lens through which we observe and attempt to understand the movies of the asymmetric and dynamic structures we call life.

## Author profile

**Peter Rosenthal** studied physics at Harvard College and then obtained a PhD in the Harvard biophysics programme working on X-ray crystallography of influenza virus surface glycoproteins. He moved to the MRC Laboratory of Molecular Biology in Cambridge to work on the development of experimental and computational methods for high-resolution structure determination by single particle electron microscopy without crystals. Since 2005, he has been a Programme Leader in the Division of Physical Biochemistry at the MRC National Institute for Medical Research at Mill Hill, London. His research applies electron microscopy to the study of biological structure from the molecular to the cellular scale including the structural analysis of lipid-enveloped viruses. In 2015, his laboratory will join the Francis Crick Institute in London.
